# Perception of National Park Soundscape and Its Effects on Visual Aesthetics

**DOI:** 10.3390/ijerph19095721

**Published:** 2022-05-08

**Authors:** Peng Wang, Chaoqun Zhang, Hesheng Xie, Wenjuan Yang, Youjun He

**Affiliations:** Research Institute of Forestry Policy and Information, Chinese Academy of Forestry, Beijing 100091, China; wpeng.up@foxmail.com (P.W.); chaoqun_zhang@outlook.com (C.Z.); hshxie@hotmail.com (H.X.); yangwenjuan31@126.com (W.Y.)

**Keywords:** landscape architecture, soundscape, perception behavior, national park of China, visual aesthetics, subjective perception

## Abstract

Soundscape perception is a very weak link in the national park landscape evaluation system in China. A thorough understanding of soundscapes and their effects on visual aesthetics is important for the management of national park landscapes. In this study, features of soundscapes (e.g., loudness, frequency, preference, and auditory satisfaction) were investigated based on 394 valid questionnaires of residents in the Qianjiangyuan National Park Pilot Area. The effects of soundscape on visual aesthetics were analyzed using the PLS-SEM. The results demonstrated that: (1) Peddling voice and insect sound were the loudest components in the soundscape, running water and birdsong were the most commonly heard and most preferred, religious sound was the quietest and least frequently heard, and horn was the least preferred. Residents in the Pilot Area were generally satisfied with the auditory environment. (2) Both sound frequency and preference have significant effects on auditory satisfaction, but preference (path coefficient = 0.426) has a larger effect than does frequency (path coefficient = 0.228). (3) Loudness has negligible effects on visual aesthetics, but other soundscape characteristics did influence visual aesthetics. Soundscape preference had the most significant effect (path coefficient = 0.305), followed by auditory satisfaction (path coefficient = 0.174), and sound frequency (path coefficient = 0.165). Among them, effects of perception frequency are the indirect utilities.

## 1. Introduction

At present, noise pollution has aroused widespread concern of the public. Studies have shown that noise exposure leads to a series of problems, such as sleep disorders with awakenings [1], learning impairment [2,3,4], hypertension ischemic heart disease [5,6], diastolic blood pressure [7], reduction in working performance [8,9], annoyance [10,11], and so on. With the emergence of the concept of soundscape, people’s attention to sound began to expand from controlling noise pollution to perceiving sound. The concept of soundscape was originally developed by R.M. Schafer, a Canadian musician, environmentalist, and educator. Schafer is credited with pioneering the study of auditory culture, and published several landmark books on soundscape, including The Tuning of the World. The International Organization for Standardization (ISO) provides with the ISO/FDIS 12913-1 2014 a clear definition to understand this innovation in acoustics. Soundscape is an “acoustic environment as perceived or experienced and/or understood by a person or people, in context [12]”. It has important ecological, cultural, and social values, and is regarded as a precious resource worthy of protection [13]. Soundscape emphasizes the perception, understanding, and reconstruction of the sound environment by individuals or societies, and is essentially an interaction between people and the sound environment [14].

Since the establishment of the soundscape concept, it has received increasing attention from scholars. Research objects include urban built-up areas [15,16], parks and green spaces [17,18], tourist destinations [19], religious sites [20], and other regions, and the soundscape has been investigated in several countries and disciplines. Research on the soundscape of national parks began in the late 1970s in the United States, and the National Park Service (NPS) has issued more than 10 laws and policies to ensure the protection of soundscape (such as The National Parks Overflights Act, National Parks Air Tour Management Act, Director’s Order 47–NPS 2000: Soundscape reservation and noise management) [21]. Pilcher et al. have found that artificial noise reduces the quality of tourists’ experience in the national park, and they have suggested developing soundscape management policies to enhance tourists’ auditory experience [22]. Weinzimmer et al. selected natural sounds as a control and studied the impact of three types of noises from propeller planes, motorcycles, and snowmobiles on the participants’ evaluation of the soundscape in national parks [23]. In addition, New Zealand, Australia, the United Kingdom, Japan, and other countries also conducted studies of soundscapes in national parks. The New Zealand Department of Resource Conservation (DOC) has formulated standards for soundscape management in different seasons and periods based on different functional zones in national parks, guiding visitors to enjoy sounds in specific areas. Researchers in Australia believe that noise caused by tourism activities is the focus of soundscape management in national parks. Watts and Pheasant selected the Scottish Highlands and Dartmoor National Park as the study area and established a visual and auditory perception prediction model (i.e., the tranquility rating prediction tool (TRAP)) to predict the tranquility of a wilderness area [24]. Japan’s research on soundscape has mainly focused on gardens, with relatively little research in national parks. The selection activity of the “Top 100 soundscapes in Japan” initiated by Japan in 1996 has played an important role in awakening the public’s awareness of soundscape heritage protection.

In recent years, more and more scholars began to pay attention to the relationship between soundscape and visual aesthetics. Schroeder and Anderson found that the evaluation of landscape aesthetics largely depends on the sounds people hear locally [25]. Carles et al. used 36 sounds and images to study the interaction between visual and auditory stimuli, and their results have shown that the consistency (or coherence) between sound and image will affect landscape preference [26]. Based on the subjective evaluation of visual and auditory interaction effects in different environments, Jeon and Jo used head-tracking technology to investigate the relationship between the overall satisfaction of landscape and soundscape. Their research has shown that the availability of visual information affects the auditory perception of many sounds, and the availability of auditory information also affects the visual perception of many landscape elements [27].

Soundscape research has represented a paradigm shift in the field of sound evaluation. It improves people’s traditional cognition of sound and expands the existing physical measurement methods [28]. Although some research has been conducted to evaluate national park soundscapes [24,29], most studies are theoretical, and only a few have analyzed public perception of soundscape and its influence on other senses. This has limited the significance of these studies in the conservation management of national park soundscapes. Soundscape has been considered in the management of national parks in Europe and the United States, but the practical application of soundscape research has been limited to classification and control. China began to establish national parks in 2015 and is in the pilot construction stage; therefore, there are few thorough studies investigating and evaluating soundscape perception and the influence of audio-visual sensory effects in national parks. Existing studies have focused on developed urban areas. Therefore, this study investigates and analyzes the features of soundscapes (e.g., loudness of, frequency of, preference for, and satisfaction with particular components of the soundscape), and explores the effects of these four perception behaviors of soundscapes on visual aesthetics in the Qianjiangyuan National Park Pilot Area (denoted as the Pilot Area) in order to provide specific methodological guidance for the management and design of soundscapes in national parks.

## 2. Materials and Methods

### 2.1. Overview of the Study Area

**Figure 1:**
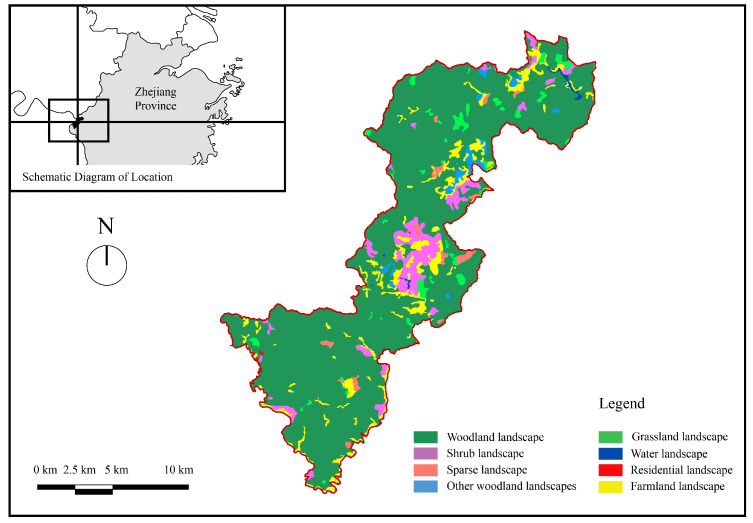
Location and land use status of the study area.

The Qianjiangyuan National Park Pilot Area is one of 10 pilot areas of the national park system in China. The pilot area is located in western Zhejiang Province (east longitude 118°01′–118°37′, north latitude 28°54′–29°30′) at the junction of Zhejiang, Anhui, and Jiangxi Provinces (Figure 1). The Pilot Area covers about 252 km^2^. The Pilot Area has the world’s most well-preserved, large-scale low-altitude mid-subtropical evergreen broad-leaved forest, which is a typical transitional zone linking plants of southern and northern China. The Pilot Area includes the 4 townships of Suzhuang, Changhong, Hetian, and Qixi, including 19 administrative villages and 72 natural villages, with a total population of 9744 people. There are no loud man-made noise sources inside the Pilot Area, such as roads, trains, aircraft, etc.

### 2.2. Questionnaire

#### 2.2.1. Object Selection

This study focuses on the auditory perception of soundscapes in the Pilot Area and requires a high degree of familiarity with the regional soundscape and a certain understanding of its overall features. Therefore, community residents who have lived in the area for many years are the best respondents. In this study, a questionnaire was used to investigate auditory perception, and the Likert 5 scale (1–5) was used to score the questionnaires. Of 416 questionnaires that were distributed, 394 were valid, and 94.71% were effective. This survey adopts the method of household survey, and the questionnaire is mainly distributed in residents’ homes and public activity places in their villages. All respondents are residents living in the Pilot Area, and the consent of all respondents was obtained before the questionnaire survey. The survey was conducted in the form of one-on-one interview because the respondents were generally older. The statistical results show that the number of male and female respondents was basically the same, as the number of male and female respondents were 191 and 203, respectively, accounting for 48.47% and 51.52% of the total, respectively. The surveyed residents were primarily middle-aged (41–55, 37.82%) and elderly (56–70, 33.50%). The majority (47.97%) of respondents had elementary school level education or less, and 63.20% had farming occupations. Most (79.19%) had an average annual income of CNY 20,000 to CNY 50,000, and 86.80% had lived in the area for >20 years (Table 1).

#### 2.2.2. Questionnaire Content

The questionnaire included four main parts. The first was basic respondent information, including gender, age, and place of residence. The second was a typical landscape survey, including sound loudness, frequency, and preference. The third was an evaluation of auditory satisfaction, including overall harmony, comfort, and satisfaction. The fourth was an assessment of the effects of soundscape on visual aesthetics, including natural, human, social, and artistic aesthetics (Appendix A).

### 2.3. Structural Equation Modeling (SEM)

#### 2.3.1. Parameters

Structural equation modeling (SEM) is a multivariate statistical method that applies linear equations to represent the relationship between latent variables and between observed and latent variables, and has outstanding advantages in the study of perception [30]. SEM provides a method to deal with measurement errors, using multiple indicators to reflect latent variables, and can estimate the relationship between the factors of the whole model. It is more accurate and reasonable than the traditional regression method [31], and has been applied to the field of soundscape research [32]. For example, Liu et al. constructed an SEM reflecting the relationship between soundscape perception and landscape evaluation, and their research results have shown that landscape satisfaction has a significant positive impact on soundscape pleasure [33]. This study includes the loudness, frequency, preference, and satisfaction, which are difficult to measure accurately and directly, and SEM can compensate for traditional statistical methods that require indirect analysis through measurable variables. Therefore, PLS-SEM was chosen to investigate the effects of soundscape perception in the Pilot Area. PLS-SEM is an analysis of variance method based on partial least squares. It is an iterative estimation method combining principal component analysis and multiple regression; it is also a causal modeling method. PLS-SEM has low requirements for sample size and sample distribution and has great advantages in landscape perception research and analysis.

When sound discomforts the body, it becomes noise, and noise deprives the person of the state of mind necessary for experiential perception. Hence, the public can experience soundscape perception only if they are satisfied with the perceived physical properties of the soundscape [34]. This paper investigates the effects of loudness, frequency, preference, auditory satisfaction, and visual aesthetics as latent variables derived from 19 variables observed in this study. The indices are constructed based on available data and existing research [33] (Table 2). Among them, the perception of soundscape loudness refers to the index of sound size felt by human ears; the sound frequency is an important index reflecting the perceptible quantity of soundscape; the sound preference is an important index to reflect people’s preference for soundscape; auditory satisfaction refers to people’s satisfaction with the auditory perception of the overall soundscape; visual aesthetics is a comprehensive evaluation index that reflects people’s visual landscape [33,35].

#### 2.3.2. Theoretical Hypotheses

**Figure 2:**
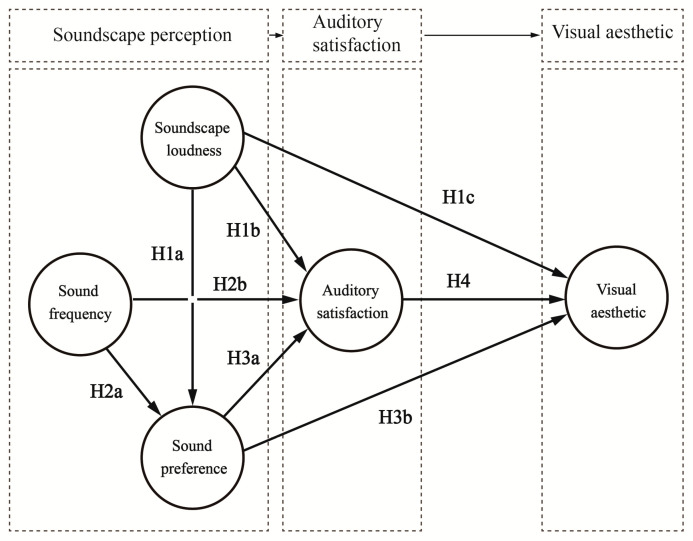
Theoretical hypotheses model.

There are differences in loudness of, frequency of, and preference for different soundscapes, and the public’s auditory and visual perception evaluation of the landscape in the Pilot Area is influenced not only by the soundscape types and sound sources, but also by the loudness of, frequency of, and preference for different sources, and their interactions. To clarify and improve the explanatory and predictive power of soundscape perception, we analyzed these factors with reference to theories related to planned behavior, proposed the hypotheses, and constructed the theoretical model of the effects of perception (Figure 2). Planned behavior theory is a theory that explains the general decision-making process of individual behavior from the perspective of information processing and the concept of expected value. This theory has been widely used in the field of behavior research and has been proven to significantly improve the explanatory and predictive power of behavior research [36].

(1) Soundscape loudness: The public’s perception of the loudness of the soundscape is mainly expressed at the level of the sound. The louder the soundscape, the clearer the sound heard by the human ear, which may increase people’s satisfaction with the soundscape. Additionally, soundscape loudness is related to the visual perception of the environment. In the interaction between auditory and visual senses, soundscape loudness may be related to visual aesthetics:

**Hypothesis** **1a** **(H1a).** *Soundscape loudness affects soundscape preference*.

**Hypothesis** **1b** **(H1b).** *Soundscape loudness affects auditory satisfaction*.

**Hypothesis** **1c** **(H1c).** *Soundscape loudness affects visual aesthetics*.

(2) Sound frequency: The frequency reflects the number of sounds of a certain type perceived by the public, and the frequency of sounds may increase preference for and satisfaction with the soundscape. At the same time, it may generate a sense of boredom and reduce satisfaction with the soundscape:

**Hypothesis** **2a** **(H2a).** *Sound frequency affects preference*.

**Hypothesis** **2b** **(H2b).** *Sound frequency affects auditory satisfaction*.

(3) Sound preference: Preference is a measure of how much people like a sound. In general, the more people like a sound, the higher their satisfaction with that sound. Meanwhile, due to the close connection between auditory and visual senses, soundscape preference may influence people’s visual perception of the environment:

**Hypothesis** **3a** **(H3a).** *Sound preference affects auditory satisfaction*.

**Hypothesis** **3b** **(H3b).** *Sound preference affects visual aesthetics*.

(4) Auditory satisfaction: In the process of perceiving soundscape, people will make visual associations, and the increase in people’s satisfaction with a certain sound may enhance the visual perception of the environment in which the sound is located:

**Hypothesis** **4** **(H4).** *Auditory satisfaction affects visual aesthetics*.

## 3. Results

### 3.1. Descriptive Statistics of Soundscape Perception

#### 3.1.1. Evaluation of Typical Soundscapes

**Figure 3:**
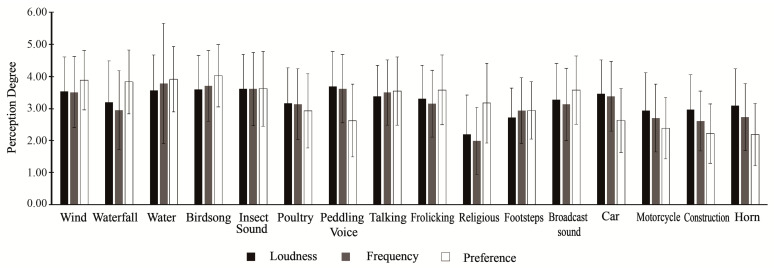
Assessment of typical soundscape perception.

Through research visits, references to existing literature [37,38], and expert consultation, 16 typical components of natural soundscapes were identified, including birdsong, insect sound, wind, running water, waterfall, poultry, talking, frolicking, peddling voice, footsteps, religious sound, broadcast sound, car, motorcycle, horn, and construction. Among them, the first six were classified as natural sounds, the middle five as sounds of human activities, and the last five as mechanical sounds. The analysis of perception of typical soundscapes was investigated primarily in terms of loudness, frequency, and preference. The results are shown in Figure 3.

Soundscape loudness is the intensity of sound perceived by the human ears and is a key indicator of the Pilot Area sound level. Peddling voice (3.69) was the loudest sound in the Pilot Area, followed by insect sound (3.62), birdsong (3.60), running water (3.57), wind (3.54), car (3.46), talking (3.38), frolicking (3.30), broadcast sound (3.27), waterfall (3.20), poultry (3.17), horn (3.09), construction (2.96), motorcycle (2.93), footsteps (2.71), and religious sound (2.19). Overall, natural sounds were the loudest (3.45), followed by mechanical sounds (3.14), and sounds of human activities (3.05).

The frequency of sound perception is an important indicator of the amount of sound in the Pilot Area. The frequency of perceiving sound perception was highest for running water (3.78), followed by birdsong (3.71), peddling voice (3.62), insect sound (3.61), wind (3.51), talking (3.50), car (3.38), frolicking (3.15), poultry (3.13), broadcast sound (3.13), waterfall (2.94), footsteps (2.93), horn (2.73), motorcycle (2.70), construction (2.61), and religious sound (1.99). Overall, natural sounds were perceived most frequently (3.45), followed by sounds of human activities (3.04) and mechanical sounds (2.91).

Sound preferences are an important indicator of people’s preference for the current soundscape. In the Pilot Area, birdsong was the most preferred sound (4.02), followed by running water (3.91), wind (3.89), waterfall (3.83), insect sound (3.62), frolicking (3.58), broadcast sound (3.57), talking (3.54), religious sound (3.17), footsteps (2.94), poultry (2.93), car (2.63), peddling voice (2.62), motorcycle (2.38), construction (2.22), and horn (2.19). Overall, natural sounds were most preferred (3.70), followed by sounds of human activities (3.17) and mechanical sounds (2.60).

#### 3.1.2. Assessment of Auditory Satisfaction

**Figure 4:**
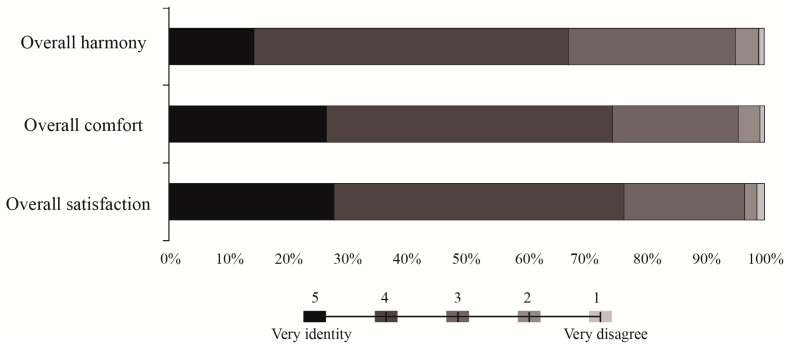
Overall assessment of auditory perception of soundscape.

In this study, the survey investigated overall harmony, comfort level, and satisfaction with soundscapes in the Pilot Area. The perception of soundscape harmony was 3.75, and 14.21% of the respondents considered soundscape coordinated while 52.79% of the respondents considered it more coordinated. Soundscape comfort was 3.96, and 26.40% of the respondents considered the soundscape very comfortable, while 47.97% of the respondents considered it more comfortable (Figure 3). This totals >70%, and suggests that most residents considered soundscapes in the Pilot Area more comfortable. Soundscape satisfaction was 3.99, and 27.66% of the respondents were very satisfied, while 48.73% of respondents were more satisfied. Overall satisfaction with soundscapes in the Pilot Area was high (Figure 4).

### 3.2. Model Results

#### 3.2.1. Model Testing

The SEM was constructed using SmartPLS 3.0 according to the above assumptions, and the corrected data were brought into the model. The primary results were:

(1) Reliability test. Internal consistency and composite reliability (CR) tests were used to assess reliability. The former was measured primarily using Cronbach’s Alpha. Except for visual aesthetics, all indices were >0.7, and visual aesthetics was nearly 0.7 (0.692) (Table 3). It is generally believed that the closer Cronbach’s Alpha is to 1, the higher the reliability is, and Cronbach’s Alpha ≥ 0.7 represents a high reliability interval [39]. The combined reliability test showed that the CR was >0.8, indicating that all variables passed the reliability test and were consistent and stable.

(2) Validity test. Discriminant validity and convergent validity were used to detect exclusivity and distribution problems. The discriminant validity is determined by measuring whether the square root of the average variance extracted values (AVE) is greater than the absolute value of the correlation coefficient of other latent variables. The convergent validity is determined by comparing the AVE value to 0.5 (when AVE > 0.5, the test is valid). Convergent (Table 3) and discriminant (Table 4) validity tests revealed that the proposed model had a rational convergent validity and reasonable discriminant validity.

(3) Significance test. A significance test was performed on the model using Bootstrapping, and the results are shown in Table 5. Hypotheses H1a, H1b, and H1c did not reach significance and did not support the original hypothesis. Hypotheses H2a, H2b, H3a, H3b, and H4 all reached significance.

#### 3.2.2. Analysis of Model Results

**Figure 5:**
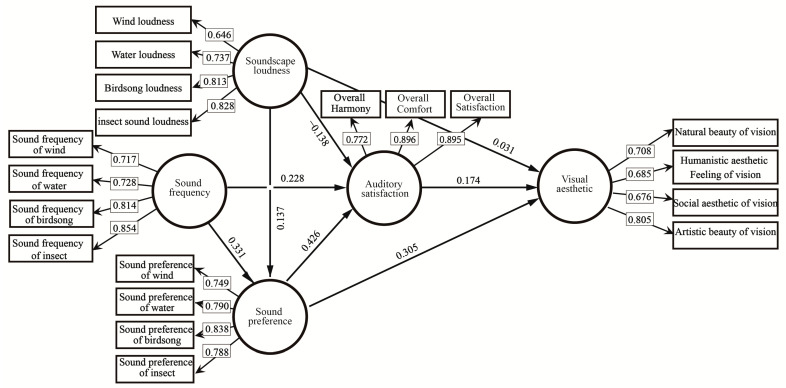
Soundscape auditory perception impact model.

The above model tests indicated that the model can account for the effects of soundscape perception. The model results are shown in Figure 5:

(1) Effects of soundscape loudness. Soundscape loudness did not significantly affect soundscape preference (*p* = 0.180), auditory satisfaction (*p* = 0.186), or visual aesthetics (0.569), and hypotheses H1a, H1b, and H1c were not supported.

(2) Effects of soundscape frequency. The frequency of sound perception significantly affected soundscape preference (*p* = 0.002) and auditory satisfaction (*p* = 0.03), supporting hypotheses H2a and H2b with path coefficients of 0.331 and 0.228, respectively. For each additional unit increase in frequency, soundscape preference and auditory satisfaction increased by 0.331 and 0.228 units, respectively.

(3) Effects of soundscape preference. Soundscape preference significantly affected auditory satisfaction and visual aesthetics, supporting hypotheses H3a and H3b with path coefficients of 0.426 and 0.305, respectively. For each additional unit of soundscape preference, auditory satisfaction and visual aesthetics increased by 0.426 and 0.305 units, respectively.

(4) Effects of auditory satisfaction. Auditory satisfaction significantly affected visual aesthetics (*p* = 0.004), supporting hypothesis H4 with a path coefficient of 0.174. For each additional unit of auditory satisfaction, visual aesthetics increased by 0.174 units.

Both sound frequency and sound preference affect auditory satisfaction, and the effect of preference (0.426) is greater than that of frequency (0.228). Of the two factors affecting visual aesthetics, preference (0.305) also had a greater effect than did auditory satisfaction (0.174). These results demonstrated the importance of preference on perception of soundscapes in the Pilot Area.

#### 3.2.3. Model Validation

Table 6 illustrates the effects of soundscape perception on visual aesthetics. Preference (0.380), satisfaction (0.174), and frequency (0.165) had significant effects on visual aesthetics, while loudness did not (*p* > 0.05). Satisfaction had a direct effect and frequency had an indirect effect, and frequency influenced visual aesthetics by influencing preference and auditory satisfaction.

## 4. Discussion

### 4.1. Features of Perception Behavior of National Park Soundscape

Peddling voice was the loudest sound (3.69) in the Pilot Area and was one of the most frequent (3.62) and least preferred sounds (preference value of 2.62), indicating that this type of sound had a strong influence on auditory perception and should be targeted for improvement by landscape management. The biggest reason for peddling voice loudness is that hawkers generally drive into the Pilot Area to sell their goods with horns on board, and the loudness of the peddling voice is high due to the loudness of the horns. The loudness of religious sounds such as temple bells was the lowest, mainly because religious buildings such as Lingyun Temple were generally far away from residences. This also led to the less frequent perception of these sounds. Liu et al. has studied that people’s perceived biological sound size has a significant positive correlation with landscape shape index, biodiversity index, and building density [40]. Some scholars have also confirmed that changes in land use have led to changes in the calls of birds and amphibians, and changes in vegetation structure have also led to the size of biological sounds [41,42,43]. Therefore, this study suggests that soundscape loudness may be related to landscape structure or land use structure.

In the Pilot Area, birdsong (3.71), running water (3.78), and wind (3.51) were the most frequently perceived sounds. These three sounds were also loud (3.60, 3.57, and 3.54, respectively) and preferred (4.02, 3.91, and 3.89, respectively), suggesting that they should be maintained and protected in the context of ecological conservation and community development of the Pilot Area. This also confirms public preference for strong natural soundscape attributes over the sounds of human activities and mechanical sounds [44]. Additionally, studies have confirmed that listening to natural sounds can reduce tension and anxiety [45,46], which is consistent with the higher preference for natural soundscapes. Birdsong and running water are often considered the most popular soundscapes [47]. The wild birds in the Pilot Area consisted of forest birds, raptors, and water birds, and the species diversity is rich, providing a rich birdsong soundscape for local residents [48,49]. Additionally, the Pilot Area is in a central subtropical warm and humid monsoon region with abundant rainfall, and many water systems [50], which is consistent with the results of soundscape perception frequency and preference in this study.

Sounds such as horns (2.19), construction (2.22), and motorcycles (2.38), which are typical mechanical sounds and stand out in the overall quiet national park sound environment [51,52,53], were least preferred. However, these sounds were heard infrequently, which indirectly confirms the high auditory satisfaction of the soundscape in the study area. The soundscape experiment conducted by Pilcher et al. in the Muir Woods National Monument has also confirmed that most tourists were very happy with the sound of wind and water: the more noise caused by human activities, the less tourists like it. They suggested strengthening the monitoring of artificial sound in the park and setting noise control indicators and quality standards [22]. Beal investigated visitors’ perception of noise at national parks in Australia and concluded that mechanical sound was the most unacceptable [54], and this study is consistent with her findings. Additionally, she also conducted a study investigating whether national parks should take measures to limit human noise, which showed that half of the people surveyed supported such measures and half of the people surveyed did not. This is an important reference for the management of national park soundscape in China, which has a high population density.

The value of harmony of various sound sources in the Pilot Area was 3.75, the comfort value was 3.96, and the satisfaction value was 3.99, showing that soundscape can make people feel comfortable and residents in the Pilot Area are overall satisfied. Weng et al. reported that landscape environments with high auditory harmony can better help people to recover psychologically in the environment [38]. Szeremeta et al. found that visitors perceive rich sound information, including volume, tone, and timbre, and that these sound contents have different degrees of influence on visitors’ satisfaction [55]. In general, in order to protect the soundscape satisfied by community residents, the delimitation of functional zoning is very important, especially the definition of the scope of human activities (such as peddling voice). The value and benefit of soundscape is the driving force to promote soundscape protection [13]. The national park management department should speed up the formulation of soundscape protection planning, formulate soundscape management objectives, element identification methods, evaluation technology, and risk management measures, and establish a soundscape monitoring system.

### 4.2. Effects of National Park Soundscape on Visual Aesthetics

Soundscape loudness has negligible effects on visual aesthetics and is instead mainly related to the amplitude of the sound source. People generally prefer soundscapes with moderate loudness, and this type of sound can play a significant role in improving people’s auditory experience. Li et al. surveyed visitors to the soundscape of Meiling National Forest Park and found that environments that are too loud can make people feel irritable, but environments that are too quiet can make people restless and nervous [56]. Liu et al. believed that among different types of soundscapes, the weaker the dominance of mechanical sound and the stronger the dominance of natural sound, the higher the pleasure of the soundscape [33]. Respondents in the Pilot Area preferred quiet to moderate natural sounds, and the preference for louder mechanical sounds was lower. There was a positive correlation between the existence of beneficial soundscape and the perceived tranquility, and the noise level was affected by the characteristics of the environment, which was consistent with the research results of an auditory and visual perception survey conducted by Cassina et al. in Italy [57]. The research conclusion of this paper indirectly confirms this point.

Soundscape preference can significantly affect the visual experience of the public, and direct utility exceeds indirect utility. Preferences for natural soundscapes (e.g., birdsong, insect sounds, and running water) affect overall auditory satisfaction with soundscapes in the Pilot Area. Meanwhile, visual aesthetic satisfaction is proportional to soundscape preference, and soundscape preference has an important influence on visual aesthetics. To some extent, visual satisfaction with a certain landscape will increase with an increase in preferred soundscape [27]. Schroeder et al. also concluded that the public’s aesthetic perception of a landscape depends to a large extent on the sound heard there [25]. Previous studies have shown that people’s perception and evaluation of soundscapes were scene dependent, that is, different environments affect users’ evaluation [58]. This is also supported by the results of a comprehensive evaluation of the audio-visual perception of landscapes with different gradients [59]. Hence, the visual landscape evaluation and the soundscape evaluation jointly determine the results of national park evaluation. This study suggests that national park soundscape preference may be directly related to the evaluation of national park landscape. In addition, previous studies proved that cultural background, age, gender, and lifestyle affect the evaluator’s visual aesthetics [60,61,62]; Yu and Kang also proved that the soundscape evaluation was affected by the respondents’ individual characteristics, social background, behavior, and psychological factors [63], and they generally prefer natural sounds and hate mechanical sounds [64], but this needs to be further supported by the demographic characteristics data of specific populations, which is also the focus of the next research.

Auditory satisfaction significantly affects visual satisfaction, and is a direct utility. When humans perceive a specific environment, multiple senses are at work simultaneously, and it is difficult to separate one perception from the others. This study demonstrates that auditory perception can largely influence visual perception via audio-visual interaction [65]. Although the auditory organ is generally considered to play a minor role in human information collection, this study combined field research and relevant earlier research to suggest that, for a special nature reserve such as a national park, the impact of soundscape in the aesthetic evaluation of integrated landscapes may exceed our empirical expectations. Morinaga et al. conducted an evaluation experiment of waterfront space using audio-visual information. They studied the interaction of audio-visual information and the relationship between the physical characteristics of underwater sound and impression perception. The results have shown that visual images have a great impact on the impression perception of waterfront space. With the increase in underwater sound level, sound is often considered more unpleasant [66]. Additionally, sound frequency can indirectly affect the visual aesthetics. These results demonstrated that the number of times people hear sounds will, to some extent, influence visual aesthetics by affecting the preference and satisfaction of people’s auditory senses [21,67].

In summary, sound frequency and preference both affect auditory satisfaction, and the effect of preference is greater than that of frequency. Among the two factors affecting visual aesthetics, preference is also more influential than is auditory satisfaction, suggesting that preference plays a key role in the Pilot Area’s landscape aesthetic quality. Based on the above analysis, it can be concluded that the more the public likes a specific soundscape, the more satisfied the auditory senses are, and the more the soundscape is consistent with its visual associations, the greater the visual aesthetic utility of the soundscape. Zhao et al. used photographs as a medium for visual aesthetic evaluation and paired them with related sounds to reach conclusions that are consistent with those of this study [68]. This study also provides direction for the design and management of national park soundscapes: national park managers should consider the negative effects of artificial soundscape loudness and set acceptable noise thresholds for mechanical sounds that may be generated in national parks. In addition, the relationship between soundscape preference, auditory satisfaction, and audio-visual aesthetics should be taken into account in planning and design in order to protect both soundscape and visual landscape [69].

### 4.3. Limitations and Future Outlook

In this study, 16 soundscapes in the Qianjiangyuan National Park Pilot Area were investigated, and auditory perception of these soundscapes were analyzed. Subject to objective conditions, this paper mainly investigated four common components of natural soundscapes to study the effects. All four of these soundscape preferences impacted auditory satisfaction and the visual aesthetic experience. There are many kinds of soundscapes (including soundscapes not involved in this study) in national parks, and each one is perceived in different ways and to different degrees. Therefore, this is a preliminary study of soundscape perception, and much work is needed to understand the role of soundscape in national park landscape management in the future. Additionally, the respondents in this study were mainly community residents living in the interior of the national park, whose demographic characteristics are somewhat more homogeneous than those of migrant groups. Therefore, whether the community residents’ auditory perceptions reflect the opinions of the general public is a question worth further discussion.

This study concluded that national park landscape aesthetic quality is influenced by the multi-sensory interaction of visual and auditory effects. Research and evaluation of national park soundscape should transition from the primary stage of traditional noise control to the stage of soundscape perception, and should include more diversified and thorough research with the help of VR, eye tracking technology, physiological response monitoring, and other new technologies [28]. In addition, it is also necessary to carry out more in-depth soundscape research in combination with real-time sound level monitoring (dBA), and strengthen the comparison of perceived data of various populations. The future management of landscapes in national parks should also go beyond the traditional visual-oriented thinking, fully explore and utilize multi-sensory elements, and include more stakeholders, so as to provide more convincing evidence for the scientific management and rational use of national parks.

## 5. Conclusions

Soundscape perception is an important but very weak link in the current national park landscape evaluation system in China, and should be strengthened. This paper used a combination of qualitative and quantitative methods to explore the characteristics of soundscape perception behavior and its influence on visual aesthetics in the Qianjiangyuan National Park Pilot Area, and concluded that: (1) The Pilot Area included six natural sounds, five mechanical sounds, and five sounds of human activities. Peddling voice has the highest loudness (3.69), and religious sound has the lowest loudness (2.19); sound frequency of water was the highest (3.78), and that of religious sound was the lowest (1.99); sound preference of birdsong was the highest (4.02), and that of horn was the lowest (2.19). Overall, the soundscapes in the Pilot Area are highly coordinated (soundscape harmony = 3.75) and comfortable (soundscape comfort = 3.96), and the local residents are satisfied with the soundscape in general (soundscape satisfaction = 3.99). (2) Both sound frequency and preference have significant effects on auditory satisfaction, and the effect of preference is direct and is greater than that of frequency. (3) Soundscape preference and auditory satisfaction can directly influence visual aesthetics, while frequency influences visual aesthetics indirectly through preference and auditory satisfaction. Preference has the largest effect size, followed by auditory satisfaction, frequency, and loudness, which had negligible effects on visual aesthetics. The results confirmed that the perception of soundscape has an impact on the visual aesthetic experience, and further expanded the influencing factors of soundscape perception from the aspects of loudness, frequency, preference, and auditory satisfaction. This study is important for the management of the national park landscapes and can provide a concrete reference for soundscape planning of national parks after the end of the China system pilot.

## Figures and Tables

**Table 1:** Demographics of residents interviewed.

Demographics		Quantity (Person)	Percentage (%)
Gender	Male	191	48.47
	Female	203	51.52
Age	≤25 years old	14	3.55
	26–40 years old	47	11.93
	41–55 years old	149	37.82
	56–70 years old	132	33.50
	≥71 years old	52	13.20
Education level	Primary school or below	189	47.97
	Junior high school	130	32.99
	High school and technical secondary school	55	13.96
	Higher vocational and junior college	17	4.31
	University and above	3	0.76
Professional	Farming	249	63.20
	Individual service	79	20.05
	Enterprise staff	28	7.11
	Migrant workers	21	5.33
	Student	8	2.03
	Other	9	2.28
Annual income	≤CNY 20,000	163	41.37
	CNY 30,000–CNY 50,000	149	37.82
	CNY 60,000–CNY 150,000	64	16.24
	CNY 160,000–CNY 300,000	13	3.30
	≥CNY 310,000	5	1.27
Years of local residence	≤5 years	10	2.54
	6–10 years	21	5.33
	11–20 years	21	5.33
	≥21 years	342	86.80
Villages and towns	Hetian township	124	31.47
	Qixi town	109	27.66
	Suzhuang town	142	36.04
	Changhong township	19	4.82

**Table 2:** Model metrics.

Latent Variables	Observed Variables	Description
Soundscape loudness	Wind loudness	Feel the loudness of the wind
	Water loudness	Feel the loudness of the water
	Birdsong loudness	Feel the loudness of the birdsong
	Insect sound loudness	Feel the loudness of the insect sound
Sound frequency	Sound frequency of wind	The frequency with which wind is perceived
	Sound frequency of water	The frequency with which water sound is perceived
	Sound frequency of birdsong	The frequency with which birdsong sound is perceived
	Sound frequency of insect	The frequency with which insect sound is perceived
Sound preference	Sound preference of wind	Degree of preference for wind
	Sound preference of water	Degree of preference for water sound
	Sound preference of birdsong	The degree of preference for birdsong
	Sound preference of insect	The degree of preference for insect sound
Auditory satisfaction	Auditory harmony	The degree of auditory harmony
	Auditory comfort	The degree of auditory comfort
	Auditory satisfaction	The degree of auditory satisfaction
Visual aesthetic	Natural beauty of vision	The visual beauty of forest, water, and other natural landscapes
	Humanistic aesthetic feeling of vision	The visual aesthetic feeling of rural and cultural landscape
	Social aesthetic of vision	The intimacy of the relationship between visual landscape and people
	Artistic beauty of vision	The artistic beauty of visual landscape

**Table 3:** Reliability and convergent validity test results.

Latent Variables	Cronbach’s Alpha	Composite Reliability (CR)	Average Variance Extracted Values (AVE)
Soundscape loudness	0.761	0.844	0.577
Sound frequency	0.785	0.861	0.609
Sound preference	0.802	0.870	0.627
Auditory satisfaction	0.818	0.892	0.734
Visual aesthetic feeling	0.692	0.811	0.519

**Table 4:** Discriminant validity test results.

	Soundscape Loudness	Sound Preference	Auditory Satisfaction	Visual Aesthetic Feeling	Sound Frequency
Soundscape Loudness	0.759				
Sound Preference	0.426	0.792			
Auditory Satisfaction	0.243	0.469	0.857		
Visual Aesthetic Feeling	0.204	0.401	0.325	0.721	
Sound Frequency	0.875	0.451	0.299	0.236	0.781

**Table 5:** Model fitting results.

Hypothesis	Relationships between Latent Variables	Standard Path Coefficient	*t*	*p*	Hypothesis Test Results
H1a	loudness → Sound preference	0.137	1.342	0.180	Not support
H1b	Soundscape loudness → Auditory satisfaction	−0.138	1.324	0.186	Not support
H1c	Soundscape loudness → Visual aesthetic	0.031	0.570	0.569	Not support
H2a	Sound frequency → Sound preference	0.331	3.065	0.002	Support
H2b	Sound frequency → Auditory satisfaction	0.228	2.177	0.030	Support
H3a	Sound preference → Auditory satisfaction	0.426	8.593	0.000	Support
H3b	Sound preference → Visual aesthetic	0.305	4.660	0.000	Support
H4	Auditory satisfaction → Visual aesthetic	0.174	2.909	0.004	Support

Note: “*t*” represents the statistical value of *t*-test; “*p*” represents probability; and *p* value reflects the possibility of an event.

**Table 6:** Effects of soundscape perception on visual aesthetics.

Path	Direct Utility	Indirect Utility	Total Utility
Soundscape loudness → sound preference	0.137	——	0.137
Soundscape loudness → auditory satisfaction	−0.138	0.058	−0.080
Soundscape loudness → visual aesthetic	0.031	0.028	0.059
Sound preference → auditory satisfaction	0.426	——	0.426
Sound preference → visual aesthetic	0.306	0.074	0.380
Auditory satisfaction → visual aesthetic	0.174	——	0.174
Sound frequency → sound preference	0.331	——	0.331
Sound frequency → auditory satisfaction	0.228	0.141	0.369
Sound frequency → visual aesthetic	——	0.165	0.165

## Data Availability

Data available on request due to restrictions.

## Appendix A

**Questionnaire:** Soundscape perception in Qianjiangyuan National Park Pilot Area.

Dear sir/Madam: Hello!

We are researchers and students from the Chinese Academy of Forestry. At present, we are conducting an investigation on the perception behavior of soundscape in Qianjiangyuan National Park Pilot Area. We promise that this survey will only be used for scientific research. Please rest assured to fill in the questionnaire. Thank you for your cooperation.

1. Investigation of basic information

(1) Gender:

☐ Male ☐ Female

(2) Age: ____years old

(3) Education level:

☐ Primary school or below ☐ Junior high school

☐ High school and technical secondary school

☐ Higher vocational and junior college

☐ University and above

(4) Professional:

☐ Farming ☐ Individual service ☐ Enterprise staff

☐ Migrant workers ☐ Student ☐ Other

(5) Annual income:

☐ ≤¥20,000 ☐ ¥30,000–¥50,000 ☐ ¥60,000–¥150,000

☐ ¥160,000–¥300,000 ☐≥¥310,000

(6) Years of local residence:

☐ ≤5 years ☐ 6–10 years ☐ 11–20 years ☐ ≥21 years

(7) Villages and towns:

☐ Hetian township ☐ Qixi town ☐ Suzhuang town ☐ Changhong township

2. Perception survey of single soundscape.

**Soundscape** **Category**	**Perceived** **Behavior Index**	**Option** **A Score of “5” to “1” Represents a Gradual Decrease in Loudness, Frequency, and Preference**
Birdsong	Loudness	☐ 5 ☐ 4 ☐ 3 ☐ 2 ☐ 1
	Frequency	☐ 5 ☐ 4 ☐ 3 ☐ 2 ☐ 1
	Preference	☐ 5 ☐ 4 ☐ 3 ☐ 2 ☐ 1
Insect Sound	Loudness	☐ 5 ☐ 4 ☐ 3 ☐ 2 ☐ 1
	Frequency	☐ 5 ☐ 4 ☐ 3 ☐ 2 ☐ 1
	Preference	☐ 5 ☐ 4 ☐ 3 ☐ 2 ☐ 1
Birdsong	Loudness	☐ 5 ☐ 4 ☐ 3 ☐ 2 ☐ 1
	Frequency	☐ 5 ☐ 4 ☐ 3 ☐ 2 ☐ 1
	Preference	☐ 5 ☐ 4 ☐ 3 ☐ 2 ☐ 1
(Running) Water	Loudness	☐ 5 ☐ 4 ☐ 3 ☐ 2 ☐ 1
	Frequency	☐ 5 ☐ 4 ☐ 3 ☐ 2 ☐ 1
	Preference	☐ 5 ☐ 4 ☐ 3 ☐ 2 ☐ 1
Waterfall	Loudness	☐ 5 ☐ 4 ☐ 3 ☐ 2 ☐ 1
	Frequency	☐ 5 ☐ 4 ☐ 3 ☐ 2 ☐ 1
	Preference	☐ 5 ☐ 4 ☐ 3 ☐ 2 ☐ 1
Poultry	Loudness	☐ 5 ☐ 4 ☐ 3 ☐ 2 ☐ 1
	Frequency	☐ 5 ☐ 4 ☐ 3 ☐ 2 ☐ 1
	Preference	☐ 5 ☐ 4 ☐ 3 ☐ 2 ☐ 1
Talking	Loudness	☐ 5 ☐ 4 ☐ 3 ☐ 2 ☐ 1
	Frequency	☐ 5 ☐ 4 ☐ 3 ☐ 2 ☐ 1
	Preference	☐ 5 ☐ 4 ☐ 3 ☐ 2 ☐ 1
Frolicking	Loudness	☐ 5 ☐ 4 ☐ 3 ☐ 2 ☐ 1
	Frequency	☐ 5 ☐ 4 ☐ 3 ☐ 2 ☐ 1
	Preference	☐ 5 ☐ 4 ☐ 3 ☐ 2 ☐ 1
Peddling Voice	Loudness	☐ 5 ☐ 4 ☐ 3 ☐ 2 ☐ 1
	Frequency	☐ 5 ☐ 4 ☐ 3 ☐ 2 ☐ 1
	Preference	☐ 5 ☐ 4 ☐ 3 ☐ 2 ☐ 1
Footsteps	Loudness	☐ 5 ☐ 4 ☐ 3 ☐ 2 ☐ 1
	Frequency	☐ 5 ☐ 4 ☐ 3 ☐ 2 ☐ 1
	Preference	☐ 5 ☐ 4 ☐ 3 ☐ 2 ☐ 1
Religious Sound	Loudness	☐ 5 ☐ 4 ☐ 3 ☐ 2 ☐ 1
	Frequency	☐ 5 ☐ 4 ☐ 3 ☐ 2 ☐ 1
	Preference	☐ 5 ☐ 4 ☐ 3 ☐ 2 ☐ 1
Broadcast Sound	Loudness	☐ 5 ☐ 4 ☐ 3 ☐ 2 ☐ 1
	Frequency	☐ 5 ☐ 4 ☐ 3 ☐ 2 ☐ 1
	Preference	☐ 5 ☐ 4 ☐ 3 ☐ 2 ☐ 1
Car	Loudness	☐ 5 ☐ 4 ☐ 3 ☐ 2 ☐ 1
	Frequency	☐ 5 ☐ 4 ☐ 3 ☐ 2 ☐ 1
	Preference	☐ 5 ☐ 4 ☐ 3 ☐ 2 ☐ 1
Motorcycle	Loudness	☐ 5 ☐ 4 ☐ 3 ☐ 2 ☐ 1
	Frequency	☐ 5 ☐ 4 ☐ 3 ☐ 2 ☐ 1
	Preference	☐ 5 ☐ 4 ☐ 3 ☐ 2 ☐ 1
Horn	Loudness	☐ 5 ☐ 4 ☐ 3 ☐ 2 ☐ 1
	Frequency	☐ 5 ☐ 4 ☐ 3 ☐ 2 ☐ 1
	Preference	☐ 5 ☐ 4 ☐ 3 ☐ 2 ☐ 1
Construction	Loudness	☐ 5 ☐ 4 ☐ 3 ☐ 2 ☐ 1
	Frequency	☐ 5 ☐ 4 ☐ 3 ☐ 2 ☐ 1
	Preference	☐ 5 ☐ 4 ☐ 3 ☐ 2 ☐ 1

3. Perception survey of auditory satisfaction.

**Problem Description**	**Option** **A Score of “5” to “1” Represents a Gradual Decrease in Harmony, Comfort and Satisfaction**.
Do you think the sound of the national park is harmonious?	☐ 5 ☐ 4 ☐ 3 ☐ 2 ☐ 1
Do you think the sound of the national park is comfortable?	☐ 5 ☐ 4 ☐ 3 ☐ 2 ☐ 1
Are you satisfied with the sound you heard in the national park?	☐ 5 ☐ 4 ☐ 3 ☐ 2 ☐ 1

4. Evaluation of the influence of sound on visual aesthetic experience.

**Index Description**	**Option** **A Score of “5” to “1” Represents the Reduction of Aesthetic Feeling and Intimacy Respectively**
The visual beauty of forest, water, and other natural landscapes	☐ 5 ☐ 4 ☐ 3 ☐ 2 ☐ 1
The visual aesthetic feeling of rural and cultural landscape	☐ 5 ☐ 4 ☐ 3 ☐ 2 ☐ 1
The intimacy of relationship between visual landscape and people	☐ 5 ☐ 4 ☐ 3 ☐ 2 ☐ 1
The artistic beauty of visual landscape	☐ 5 ☐ 4 ☐ 3 ☐ 2 ☐ 1

## References

[1] Muzet A. (2007). Environmental noise, sleep and health. Sleep Med. Rev..

[2] Zacarías F.F., Hernández R., Cueto J.L., Lopez S.L., Alonso-Ojembarrena A. (2013). Noise Exposure in Preterm Infants Treated with Respiratory Support Using Neonatal Helmets. Acta Acust. United Acust..

[3] Minichilli F., Gorini F., Ascari E., Bianchi F., Coi A., Fredianelli L., Licitra G., Manzoli F., Mezzasalma L., Cori L. (2018). Annoyance Judgment and Measurements of Environmental Noise: A Focus on Italian Secondary Schools. Int. J. Environ. Res. Public Health.

[4] Erickson L.C., Newman R.S. (2017). Influences of Background Noise on Infants and Children. Curr. Dir. Psychol. Sci..

[5] Dratva J., Phuleria H.C., Foraster M., Gaspoz J.M., Keidel D., Künzli N., Liu L.S., Pons M., Zemp E., Gerbase M.W. (2012). Transportation Noise and Blood Pressure in a Population-Based Sample of Adults. Environ. Health Perspect..

[6] Babisch W., Beule B., Schust M., Kersten N., Ising H. (2005). Traffic noise and risk of myocardial infarction. Epidemiology.

[7] Petri D., Licitra G., Vigotti M.A., Fredianelli L. (2021). Effects of Exposure to Road, Railway, Airport and Recreational Noise on Blood Pressure and Hypertension. Int. J. Environ. Res. Public Health.

[8] Vukić L., Mihanović V., Fredianelli L., Plazibat V. (2021). Seafarers’ Perception and Attitudes towards Noise Emission on Board Ships. Int. J. Environ. Res. Public Health.

[9] Rossi L., Prato A., Lesina L., Schiavi A. (2018). Effects of low-frequency noise on human cognitive performances in laboratory. Build. Acoust..

[10] Miedema H., Groothuis-Oudshoorn C.G. (2001). Annoyance from transportation noise: Relationships with exposure metrics DNL and DENL and their confidence intervals. Environ. Health Perspect..

[11] Licitra G., Fredianelli L., Petri D., Vigotti M.A. (2016). Annoyance evaluation due to overall railway noise and vibration in Pisa urban areas. Sci. Total Environ..

[12] (2014). Acoustics Soundscape Part 1: Definition and Conceptual Framework.

[13] Dumyahn S.L., Pijanowski B.C. (2011). Soundscape conservation. Landsc. Ecol..

[14] Zappatore M., Longo A., Bochicchio M.A. (2017). Crowd-sensing our smart cities: A platform for noise monitoring and acoustic urban planning. J. Commun. Softw. Syst..

[15] Verma D., Jana A., Ramamritham K. (2020). Predicting human perception of the urban environment in a spatiotemporal urban setting using locally acquired street view images and audio clip. Build. Environ..

[16] Ma K.W., Mak C.M., Hai M.W. (2021). Effects of environmental sound quality on soundscape preference in a public urban space. Appl. Acoust..

[17] Zhao W., Li H., Zhu X., Ge T. (2020). Effect of Birdsong Soundscape on Perceived Restorativeness in an Urban Park. Int. J. Environ. Res. Public Health.

[18] Soares A.L., Coelho J.B. (2016). Urban park soundscape in distinct sociocultural and geographical contexts. Noise Mapp..

[19] Zuo L., Zhang J., Zhang R.J., Zhang Y.Y., Hu M., Zhuang M., Liu W. (2020). The Transition of Soundscapes in Tourist Destinations from the Perspective of Residents’ Perceptions: A Case Study of the Lugu Lake Scenic Spot, Southwestern China. Sustainability.

[20] Jeon J.Y., Hwang I.H., Hong J.Y. (2014). Soundscape evaluation in a Catholic cathedral and Buddhist temple precincts through social surveys and soundwalks. J. Acoust. Soc. Am..

[21] Miller N.P. (2008). US National Parks and management of parks: A review. Appl. Acoust..

[22] Pilcher E.J., Newman P., Manning R.E. (2009). Understanding and Managing Experiential Aspects of Soundscapes at Muir Woods National Monument. Environ. Manag..

[23] Weinzimmer D., Newman P., Taff D., Benfield J., Lynch E., Bell P. (2014). Human Responses to Simulated Motorized Noise in National Parks. Leis. Sci..

[24] Watts G.R., Pheasant R.J. (2015). Tranquillity in the Scottish Highlands and Dartmoor National Park-The importance of soundscapes and emotional factors. Appl. Acoust..

[25] Schroeder H.W., Anderson L.M. (1984). Perception of personal safety in urban recreation sites. J. Leis. Res..

[26] Carles J.L., Barrio I.L., de Lucio J.V. (1999). Sound influence on landscape values. Landsc. Urban Plan..

[27] Jeon J.Y., Jo H.I. (2020). Effects of audio-visual interactions on soundscape and landscape perception and their influence on satisfaction with the urban environment. Build. Environ..

[28] Kang J., Schulte-Fortkamp B. (2015). Soundscape and the Built Environment.

[29] Taff D., Newman P., Lawson S.R., Bright A., Marin L., Gibson A., Archie T. (2014). The role of messaging on acceptability of military aircraft sounds in Sequoia National Park. Appl. Acoust..

[30] Bollen K.A. (1989). Structural Equations With Latent Variables.

[31] Kaplan D. (2000). Structural Equation Modeling: Foundations And Extensions.

[32] Hong J.Y., Jeon J.Y. (2015). Influence of urban contexts on soundscape perceptions: A structural equation modeling approach. Landsc. Urban Plan..

[33] Liu J., Yang L., Zhang X.W. (2019). Study on the relationship between sound scene perception and landscape evaluation of historical blocks—A case study of Sanfang and Qixiang in Fuzhou. Chin. Landsc. Archit..

[34] Qiu M.Y., Wang F., Sha R., Hou G.L. (2013). Tourists’ Perception of and Satisfaction with Soundscape Properties in Tourist Areas: A Case Study of Nanjing Confucius Temple-Qinhuai Scenic Area. Tour. Trib..

[35] Chen Y.X., Zhang G.F. (2021). Simulation of Optimal Frequency Regulation Control Method in Sound Scene. Comput. Simul..

[36] Duan W., Jiang G.R. (2008). Review of planned behavior theory. Prog. Psychol. Sci..

[37] Pijanowski B.C., Farina A., Gage S.H., Dumyahn S.L., Bernie L.K. (2011). What is soundscape ecology? An introduction and overview of an emerging new science. Landsc. Ecol..

[38] Weng Y.X., Zhu Y.J., Dong J.Y., Wang M.H., Dong J.W. (2021). Effects of Soundscape on Emotion and Attention on Campus Green Space—A Case Study of Fujian Agriculture and Forestry University. Chin. Landsc. Archit..

[39] Bai J.D., Liu J.C., Chen W.H. (2019). Influence on forest ecological security based on a structural equation model. Acta Ecol. Sin..

[40] Liu J., Kang J., Luo T., Behm H., Coppack T. (2013). Spatiotemporal variability of soundscapes in a multiple functional urban area. Landsc. Urban Plan..

[41] Mockford E.J., Marshall R.C. (2009). Effects of urban noise on song and response behaviour in great tits. Proc. R. Soc. B Biol. Sci..

[42] Warren P.S., Katti M., Ermann M., Brazel A. (2006). Urban bioacoustics: It’s not just noise. Anim. Behav..

[43] Joo W., Gage S.H., Kasten E.P. (2011). Analysis and interpretation of variability in soundscapes along an urban–rural gradient. Landsc. Urban Plan..

[44] Zhang M., Kang J. (2007). Towards the evaluation, description, and creation of soundscapes in urban open spaces. Environ. Plan. B Plan. Des..

[45] Ratcliffe E., Gatersleben B., Sowden P.T. (2016). Associations with bird sounds: How do they relate to perceived restorative potential?. J. Environ. Psychol..

[46] Hume K., Ahtamad M. (2013). Physiological responses to and subjective estimates of soundscape elements. Appl. Acoust..

[47] Yang M., Kang J. (2013). Psychoacoustical evaluation of natural and urban sounds in soundscapes. J. Acoust. Soc. Am..

[48] Xu D.Y., He Y.J., Zhao X.D., Ye B. (2019). Exploration on adaptive governance of Natural resources: A case study of qianjiangyuan National Park pilot System. World Agric..

[49] Qian H.Y., Yu J.P., Shen X.L., Ding P., Li S. (2019). Diversity and composition of birds in the Qianjiangyuan National Park pilot. Biodivers. Sci..

[50] Fan H.J., Wu H.Y., Zhang S.G., Li D.F. (2017). Hydrological Computation and Analysis of Majinxi Comprehensive Control Project. J. Zhejiang Univ. Water.

[51] Mennitt D.J., Fristrup K.M. (2016). Influence Factors and Spatiotemporal Patterns of Environmental Sound Levels in the Contiguous United States. Noise Control Eng. J..

[52] Krause B., Gage S.H., Joo W. (2011). Measuring and interpreting the temporal variability in the soundscape at four places in Sequoia National Park. Landsc. Ecol..

[53] Downing J.M., Stusnick E. (2000). Measurement of the natural soundscape in national parks. J. Acoust. Soc. Am..

[54] Beal D.J. (1994). Campers’ Attitudes to noise and regulation in Queensland national park. Aust. Park Recreat..

[55] Szeremeta B., Zannin P.H.T. (2009). Analysis and evaluation of soundscape in public parks through interview and measurement of noise. Sci. Total Environ..

[56] Li H., Wang Y.Q., Chen F.P. (2018). Evaluation of Tourist Survey of Soundscape in Meiling National Forest Park. Sci. Silvae Sin..

[57] Cassina L., Fredianelli L., Menichini I., Chiari C., Licitra G. (2017). Audio-Visual Preferences and Tranquillity Ratings in Urban Areas. Environments.

[58] Ma H., Wang D. (2012). Sound landscape elements of urban parks and their preliminary quantitative analysis. Noise Vib. Control.

[59] Gan Y.H., Luo T., Zhang T.H., Zhang T., Qiu Q.Y. (2013). Changes of Visual and Acoustic Landscape Along Urban-rural Gradients and Their Influence on Landscape Evaluation. Environ. Sci. Technol..

[60] Molnarova K.J., Sklenicka P., Stiborek J., Svobodova K., Salek M., Brabec E. (2012). Visual preferences for wind turbines: Location, numbers and respondent characteristics. Appl. Energy.

[61] Lindemann-Matthies P., Briegel R., Schüpbach B., Junge X. (2010). Aesthetic preference for a Swiss alpine landscape: The impact of different agricultural land-use with different biodiversity. Landsc. Urban Plan..

[62] Berg A., Koole S.L. (2006). New wilderness in the Netherlands: An investigation of visual preferences for nature development landscapes. Landsc. Urban Plan..

[63] Yu L., Kang J. (2010). Factors influencing the sound preference in urban open spaces. Appl. Acoust..

[64] Kang J., Zhang M. (2010). Semantic differential analysis of the soundscape in urban open public spaces. Build. Environ..

[65] Shams L., Kamitani Y., Shimojo S. (2002). Visual illusion induced by sound. Cogn. Brain Res..

[66] Morinaga M., Aono S., Kuwano S., Kato T. (2003). Psychological Evaluation of Waterside Space Using Audio-Visual Information. Empir. Stud. Arts.

[67] Wang R.H., Zhao J.W. (2019). A good sound in the right place: Exploring the effects of auditory-visual combinations on aesthetic preference. Urban For. Urban Green..

[68] Zhao J.W., Yang S.L., Zhang L. (2017). The effect of sound landscape on visual aesthetic perception. Urban Probl..

[69] Fowler M.D. (2013). Soundscape as a design strategy for landscape architectural praxis. Des. Stud..

